# The role of hypoglycemic phytochemicals in improving male reproductive dysfunction: a systematic review of preclinical evidence

**DOI:** 10.3389/fendo.2026.1814324

**Published:** 2026-07-13

**Authors:** Geoffrey Ayebazibwe, Herbert Izo Ninsiima, Immaculate Mandera, Lynnette Tumwine Kyokunda, Denis Okello, Daniel Matovu

**Affiliations:** 1 Department of Pharmacology, School of Medicine, Kabale University, Kampala, Uganda; 2 Departmental of Human Physiology, School of Medicine, Kabale University, Kampala, Uganda; 3 Departmental of Nursing Science, School of Medicine, Kabale University, Kampala, Uganda; 4 Department of Pathology, School of Medicine, Kabale University, Kampala, Uganda; 5 Department of Biological Sciences, Faculty of Science, Kabale University, Kampala, Uganda

**Keywords:** hypoglycemic phytochemicals, male infertility, preclinical models, sperm parameters, testosterone, type 2 diabetes

## Abstract

Diabetes mellitus (DM), which affects over 500 million individuals (7%) globally, is associated with male reproductive dysfunction in more than 15% of affected individuals. The preclinical mechanisms by which hypoglycemic phytochemical compounds alleviate reproductive dysfunction in this population are scattered. A detailed systematic review of the preclinical evidence, with a focus on mechanisms of action and application potential, is currently necessary. The objectives were to analyze preclinical evidence on the therapeutic potential and mechanistic pathways through which hypoglycemic phytochemicals improve male reproductive function in diabetes mellitus.

**Methodology:**

The protocol was registered in PROSPERO prior to screening, and PRISMA guidelines 2020 were followed. Papers included in the search were published from October 2010 to October 2025 and were identified using the PubMed, Connecting Repositories, and Google Scholar databases.

**Results:**

Only 35 studies published between 2012 and 2025 that investigated improvements in male reproductive dysfunction in preclinical settings were reviewed.

**Discussion:**

The hypoglycemic phytochemicals improved diabetes-induced reproductive dysfunction through their effects on oxidative stress, sperm parameters, spermatogenesis, testicular morphology, testosterone, follicle-stimulating hormone, and luteinizing hormone. There were variations in the outcomes measured and methodologies. The majority of hypoglycemic phytochemicals reduced reactive oxygen species, which may be inadequate in humans, where there is also a psychological impact on erection.

**Conclusion:**

The review showed that hypoglycemic phytochemicals have the potential to improve male reproductive dysfunction in preclinical models of diabetes mellitus. The synthesis highlights the beneficial effects on glycemic control, sperm quality, hormonal regulation, oxidative stress, and testicular histology via integrated metabolic and reproductive mechanisms. Standardized phytochemicals should be used to study efficacy and safety before clinical trials.

**Systematic review registration:**

https://www.crd.york.ac.uk/PROSPERO/view/CRD420251230781, identifier CRD420251230781.

## Background and rationale

Chronic hyperglycemia, systemic inflammation, and insulin resistance are characteristic of diabetes mellitus (DM) (1). DM affects over 500 million individuals (7%) globally, and the prevalence of associated male reproductive dysfunction is more than 15% (2). Evidence indicates that T2DM contributes to male reproductive dysfunction, including poor sperm quality and changes in testicular histology. The derangement of hormonal profile occurs through mechanisms such as oxidative stress and endocrine dysfunction, with research focusing mainly on male infertility involving pathologies that prevent pregnancy in animal models (3, 4).

Hypoglycemic phytochemicals are naturally occurring plant compounds that lower blood sugar levels through several distinct mechanisms of action (5). Some plant-derived phytochemical extracts, including berberine, curcumin, and *Ficus Sycomorus*, possess protective roles in animal and human male reproduction (6–8). A detailed systematic review of the preclinical importance of plant phytochemical compounds in improving reproductive function in DM, with a focus on mechanisms of action and application potential, is currently necessary.

This will help improve the quality of life for many affected people worldwide, especially in developing countries such as Uganda (9). The systematic review will inform health workers and researchers about the therapeutic benefits and limitations of hypoglycemic phytochemicals for male reproductive dysfunction associated with DM, while also helping to identify priorities for translational research.

## Research question

Primary question:

What is the role of hypoglycemic phytochemicals in improving male reproductive dysfunction in preclinical models of DM?

### Objectives

1. To analyze preclinical (*in vivo*) evidence on the effect of hypoglycemic phytochemicals on improving male reproductive function in DM.
2. To assess the mechanistic pathways (antioxidant, anti-inflammatory, hormonal regulation, and metabolic modulation) by which hypoglycemic phytochemicals affect reproductive outcomes in DM.

## Methodology

### Study design

**Figure 1:**
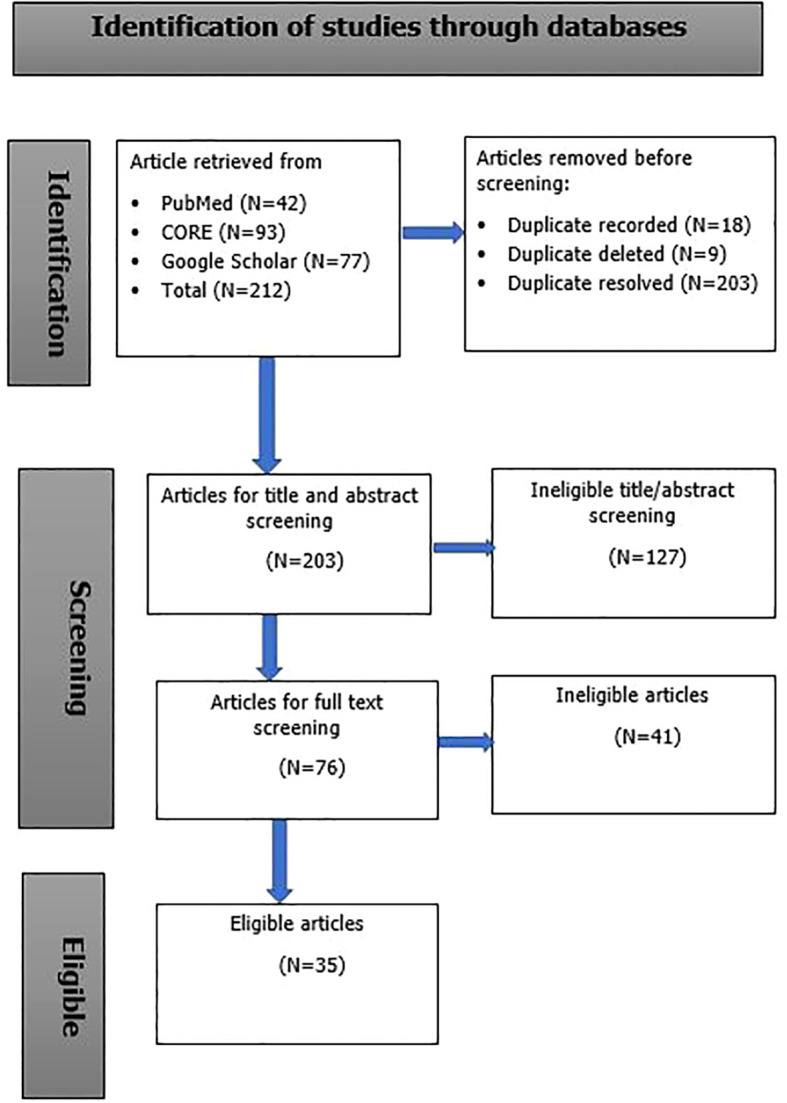
PRISMA flow diagram.

A protocol for the systematic review was published on 22 December 2025 at 11:54 UTC (Version 1.0) in PROSPERO prior to screening, with registration number CRD420251230781. Available from https://www.crd.york.ac.uk/PROSPERO/view/CRD420251230781. The review was conducted in accordance with the PRISMA 2020 guidelines. as shown in **Figure 1**.

### Search strategy

Research articles published from October 2010 to October 2025 were identified by searching the PubMed, Connecting Repositories (CORE), and Google Scholar databases. The search terms combined keywords for phytochemicals/plant extracts and hypoglycemic activity with male reproductive endpoints and type 2 diabetes mellitus (T2DM) using Boolean operators and MeSH terms: ((hypoglycemic agents[MeSH] OR hypoglycemic* OR antidiabetic* OR “blood glucose lowering”) AND (phytochemical* OR plant extract* OR botanical* OR polyphenol* OR flavonoid* OR alkaloid* OR terpenoid*)) AND ((“Male infertility”[MeSH] OR reproductive dysfunction OR infertility OR spermatogenesis OR sperm* OR testicular dysfunction) AND (male OR testis OR testes)) AND (Animals[MeSH] OR rat* OR mouse OR rodent* OR *in vivo*). No language filters were used during the search. The retrieved papers were exported to an Excel sheet for further management. Due to the abundance of publications on the subject matter, grey literature and manual searches were not performed. However, we used CORE, a connecting repository that is the largest collection of full open-access papers from repositories and journals.

### Eligibility criteria

Population: male animal models with DM.

Interventions: isolated phytochemicals or standardized plant extracts with hypoglycemic activity.

Comparators: placebo, hypoglycemic agents, or standard antidiabetic therapies.

Outcomes: Improved glucose control and fertility.

Study types: *in vivo* controlled preclinical studies.

### Defined scope

#### Inclusion criteria

- Studies using preclinical animal models involving male animals with DM only.
- Studies evaluating isolated phytochemicals or standardized plant extracts with documented hypoglycemic properties.
- Studies reporting outcomes including sperm parameters (count, motility, and morphology), male gonad hormones, testicular histology, oxidative/inflammatory biomarkers, and pregnancy outcomes.
- Studies employing both type 1 and type 2 diabetic models.

#### Exclusion criteria

- Systematic reviews of preclinical animal models involving male animals with DM.
- Uncontrolled case reports without comparative data.

### Data extraction

Data were extracted independently by two reviewers, who captured study design, sample characteristics, compound, dose, duration, outcomes, and findings. Consensus was used to resolve discrepancies. Two reviewers were involved independently, and any disagreements were resolved by a third reviewer. The inter-reviewer agreement was 0.79 (Cohen’s Kappa). Discrepancies were resolved, and the third reviewer made the final decision when consensus was not reached.

### Quality assessment

The SYRCLE Risk of Bias (RoB) tool was used to appraise the animal studies. For the overall evidence of certainty, the GRADE framework was applied. SYRCLE was used to independently assess each study across different domains, including selection, performance, detection, attribution, reporting, and judgment biases. Using RoB, the different studies were rated as low, unclear, and high risk across these domains.

### Data synthesis

Study characteristics, antioxidant activity, anti-inflammatory effects, hormonal quantities, and outcomes were used for the narrative synthesis. A meta-analysis was not performed due to heterogeneity in diabetic models, fertility function tests, and key outcomes such as sperm count, motility, and hormonal levels.

## Results

The systematic review comprised 35 studies published between 2012 and 2025 that investigated hypoglycemic phytochemicals for improving male reproductive dysfunction in preclinical settings (**Figure 1**). All the studies were preclinical experimental *in vivo* animal studies, employing normal Wistar rats, Sprague-Dawley rats, mice, and streptozotocin (STZ)- or alloxan-induced diabetic models, in addition to one study that used male rabbits (bucks) to explore the male reproductive system. Chronologically, the research output showed a steady rise over the years: 2012 (two studies; 5.71%), 2013 (one study; 2.86%), 2015 (two studies; 5.71%), 2017 (four studies; 11.43%), 2018 (four studies; 11.43%), 2019 and 2020 (three studies each; 8.57%), while2023 (one study; 2.86%), and 2021, 2024, and 2025 (four studies each; 11.43%). Thus, the peak publication years had four studies each: 2017, 2018, 2021, 2024, and 2025. These studies increasingly evaluated testosterone levels, testicular histology, and oxidative stress parameters.

### Study characteristics

The studies utilized a range of plant extracts, with the majority using leaves (80%), followed by seeds (8.57%), roots (5.71%), and stem bark and fruit peels (2.9% each). The most commonly used pharmacological comparators were glibeclamide (20%), metformin (14.29%), normal saline (8.57%), sildenafil (8.57%), insulin and distilled water (5.71% each), whereas clomiphene, cisplatin, etoposide, BPA (2,2-Di (4-hydroxyphenyl) propane), caffeine, highly active antiretroviral therapy (HAART), and metaglomide were each used in 2.86% of the studies.

**Table 1**. Study durations ranged from 14 days to 84 days, with the majority of preclinical studies lasting 28–60 days. Geographically, research was distributed across several regions: Iran (10 studies; 28.57%), Nigeria (7 studies; 20.00%), Egypt (3 studies; 8.57%), and Pakistan, South Africa, and Indonesia (2 studies each; 5.71%), whereas India, Portugal, China, Malaysia, the United states of America, Brazil, Tunisia, Poland, and Russia each contributed one study (2.86%).

**Table 1:** Study characteristics.

STUDY ID	YEAR	AUTHOR	COUNTRY	STUDY DESIGN	SAMPLE SIZE	POPULATION	INTERVENTION	COMPARATOR	PRIMARY OUTCOME	SECONDARY OUTCOME	KEY RESULTS	EFFECT SIZE	DURATION	RISK BIAS
1	2017	(10)	Malaysia	Experimental (*in vivo* animal study)	42 rats	Male rats aged 8 weeks (120–200 g)	*Gynura procumbens* Leaf	Metformin	FBS	Testosterone	Low fasting blood sugar (FBS) and increased testosterone	(p<0.05)	42 days	Low
2	2018	(11)	Nigeria	Experimental (*in vivo* animal study)	60 rats	30 male and 30 female adult Sprague-Dawley rats with Cisplatin	*Cochlospermum planchonii* Rhizome	Normal saline 2 ml/kg	Sperm parameters	Testosterone	Improved sperm parameters and testosterone	(p<0.05)	14 days	Unclear
3	2013	(12)	Portugal	Experimental (*in vivo* animal study)		Two-day-old male Wistar rats with STZ	White tea (*Camellia sinensis*)	Drinking water	Sperm parameters	Glucose tolerance	Improved glucose tolerance and Sperm paramaters	(p<0.05)	60 days	Unclear
4	2019	(11)	South Africa	Experimental (*in vivo* animal study)	60 rats	60 streptozotocin (STZ)-induced diabetic Wistar rats	Kolaviron (KV) extract from *Garcinia kola* (“bitter kola”)	Subcutaneous insulin	FBS level	Lipid peroxidation	FBS level	(p<0.05)	42 days	Low
5	2018	(13)	Nigeria	Experimental (*in vivo* animal study)	80 rats	48 mature male and 42 mature female Albino Wistar rats	*Afzelia africana (Smith)*	Glibenclamide	Libido score	Testosterone	Libido score and testosterone level	(p<0.05)	21 days	Low
6	2024	(14)	Pakistan	Experimental (*in vivo* animal study)	36 rats	18 male and 18 female adult Wistar rats’ weight 160-180g	*Prunus amygdalas*	Etoposide	Reproductive indices of rats	Hormonal parameters	Improved fertility and hormonal profile	(p<0.05)	30 days	Low
7	2021	(15)	Indonesia	Experimental (*in vivo* animal study)	48 male mice	48 Alloxan induced diabetic mice	Hesperetin flavonoids from orange peels	Metformin	Blood sugar levels	Spermatozoa quality	Decreased blood glucose levels, increased spermatozoa quantity	(P<0.05)	56 days	Low
8	2018	(16)	Iran	Experimental (*in vivo* animal study)	35 rats	28 STZ-induced diabetic male rats and 7 non diabetic rats	*Stevia rebaudiana Bertoni*	Distilled water	Blood glucose levels	Sexual dysfunction, Testosterone levels	Improved sexual dysfunction, Testosterone levels	(P<0.05)	28 days	Low
9	2021	(17)	Iran	Experimental (*in vivo* animal study)	48 rats	48 healthy male Wistar rats with an average weight of 240 g	*Securigera securidaca* seeds	Glibenclamide	Testicular tissue and energy homeostasis	Sperm parameters	Improved sperm parameters, testicular tissue and energy homeostasis	(P<0.05)	35 days	Low
10	2020	(18)	Iran	Experimental (*In vivo* animal study)	60 rats	60 STZ treated eight-week-old male Wistar rats, weighing from 200 to 250 g,	*Rosa canina*	Metformin		Levels of DNA Methyl Transferases (DNMTS) 1 and 3α	Improved blood glucose and levels of DNMTS 1 and 3α	(p <0.05)	56 days	Low
11	2017	(19)	South Africa	Experimental (*in vivo* animal study)	62 Rats	62 adult Male Sprague-Dawley rats (189.0 ± 4.5 g) STZ-nicotinamide-induced diabetes	*Hypoxis hemerocallidea* (HH) aqueous extract	(Highly Active Anti-Retroviral Therapy)HAART cocktail (zidovudine, lamivudine and nevirapine)	Testosterone levels	Expression of androgen receptors	Testosterone levels, expression of androgen receptors	(p < 0.0001)	56 days	unclear
12	2025	(20)	Iran	Experimental (*in vivo* animal study)	25 rats	25 male Wistar STZ- induced diabetic rats	*Trifolium pratense*		Blood glucose levels	Nitric oxide (NO)	FBS and (Nitric Oxide) NO while increasing c- peptide	p < 0.001	28 days	Unclear
13	2021	(21)	Iran	Experimental (*in vivo* animal study)	28 rats	28 male Wistar rats (Weight 200−250 g)	*Juglans regia L*. leaf extract	Saline	Blood glucose levels	Oxidative stress biochemical parameters	Improved blood glucose and decreased MDA and increased GSH, CAT and SOD activities	(p <0.001)	56 days	Low
14	2023	(22)	Iran	Experimental (*in vivo* animal study)	40 rats	40 Alloxan-induced male rats	Vincamine	Clomiphene	Blood sugar levels	Oxidative stress biochemical parameters	Improved FBS, oxidative stress, seminal analysis, and histological examination of the testis	(P < 0.001)	14 days	Low
15	2015	(23)	China	Experimental (*in vivo* animal study)	50 rats	50 eight-week-old Sprague-Dawley rats treated with STZ	Antioxidant Icariside II	Insulin	Blood sugar levels	Erectile parameters	Improved blood sugar, erectile parameters, cytological components and biochemistry	P < 0.05	42 days	Low
16	2020	(24)	Egypt	Experimental (*in vivo* animal study)	42 rats	42 STZ (35mg/kg) in male albino rats	Green coffee arabica extract	Metformin	Levels of testosterone	Parameters of oxidative stress	Improved testosterone levels, and oxidative stress	p<0:05	28 days	Low
17	2012	(25)	India	Experimental (*in vivo* animal study)	36 rats	36 Albino rats STZ-induced	*Mucuna pruriens (Linn.)*	Sildenafil citrate	Sexual behavior	Sperm parameters	Improved sexual behavior and Sperm parameters	P < 0.05	60 days	Low
18	2019	(26)	Brazil	Experimental (*in vivo* animal study)	20 rats	20 adult male Wistar rats	Alcohol extract of *Bauhinia forficata*	Saline solution (9%)	Histopathological analysis	Testicular morphometric,and spermatogenickinetics	No Histopathological analysis, testicular morphometric, spermatogenic kinetics	(p>.05)	30 days	Low
19	2025	(6)	Poland	Experimental (*in vivo* animal study)	25 rats	25 Alloxan-induced diabetes rats	*Mimosa pudica Linn*	Sildenafil citrate	Sperm and the histocellular arrangement of the testes	Levels of hormones (testosterone, follicle-stimulating hormone (FSH) and luteinizing hormone (LH)	Improved sperm histology and hormonal levels	Mean ± standard deviation	49 days	Low
20	2015	(27)	Iran	Experimental (*in vivo* animal study)	56 mice	56 adult male mice	*Arctium lappa* (burdock) root	Glibenclamide	Sperm parameters	Hormonal parameters	Improved sperm profile and hormonal profile	(P < 0.01)	28 days	Low
21	2021	(28)	Egypt	Experimental (*in vivo* animal study)	50 rats	50 STZ-induced rats	*Launaea nudicaulis* Ethanolic Extract	Glibenclamide	Blood glucose levels	Oxidative stress biochemical parameters	Improved blood sugar, and oxidative stress	(p ≤ 0.05)	35 days	Low
22	2022	(29)	Iran	Experimental (*in vivo* animal study)	28 rats	28 male rats STZ-induced	*Portulaca oleracea*		Hormonal profile	Oxidative stress biochemical parameters	Improved hormonal profile and oxidative stress	(P < 0.01)	56 days	unclear
23	2024	(30)	Russia	Experimental (*in vivo* animal study)	100 rats	40 male and 60 female rats	*Chicory (Cichorium intybus L.)*	Tap water	Testosterone levels	Sperm parameters and offspring outcome	Improved testosterone levels, sperm parameters, and offspring outcome	(p < 0.05)	60 days	Low
24	2024	(31)	USA	Experimental (*in vivo* animal study)	36 rats	36 adult male Wistar rats STZ-induced diabetic	Chicory (*Chicorium Intybus*) and Purslane (*Portulaca oleracea*)	Streptozotocin	Blood glucose level	Oxidative stress biochemical parameters	Improve blood sugar levels and oxidative stress	(p < 0.05)	30 days	Low
25	2022	(32)	Iran	Experimental (*in vivo* animal study)	60 rats	60 adult male Wistar rats with STZ	*Zataria multiflora Boissis*	Glibenclamide	Sperm parameters	Oxidative stress biochemical parameters	Improved sperm parameters and oxidative stress	(p <0.001)	28 days	Low
26	2025	(33)	Egypt	Experimental (*in vivo* animal study)	24 rats	24 male Wistar rats	*Momordica charantia*	Cisplatin	Oxidative stress parameters	Histology of the testis	Improved oxidative status, testis histology, and Sperm parameters	(p<0.05)	42 days	Low
27	2022	(34)	Iran	Experimental (*in vivo* animal study)		Adult male Wistar rats with STZ	*Trifolium pratense* extract	Normal saline	Oxidative stress parameters	Histology of the testis	Improved antioxidant status, sperm characteristics, testicular tissue changes, and testosterone levels	(p ≤ 0.05)	21 days	Unclear
28	2018	(35))	Nigeria	Experimental (*in vivo* animal study)	40 rats	40 adult male Wistar rats with STZ (60 mg/kg)	*Loranthus micranthus*	Gilbenclamide	Blood glucose level	Oxidative stress biochemical parameters	Improved blood glucose, hormonal profile, oxidative stress parameters,	(p< 0.05)	14 days	Low
29	2024	(36)	Indonesia	Experimental (*in vivo* animal study)	30 rats	30 male Alloxan induced rats 150–200 g	Porang (*Amorphophallus oncophyllus)*	Metformin	Spermatogenic histological criteria	Blood glucose levels	No histopathological analysis, and blood glucose	(p >0.05)	14 days	Low
30	2017	(37)	Nigeria	Experimental (*in vivo* animal study)	30 rats	30 healthy male albino rats	Soursop (*A. muricata*) Tea	Caffeine	Superoxide dismutase (SOD) and glutathione peroxidase (GPx)	Catalase (CAT) and malondialdehyde (MDA)	Improved oxidative stress parameters	(P = .05)	65 days	Low
31	2012	(25)	Nigeria	Experimental (*in vivo* animal study)	48 rats	48 Alloxan-induced diabetes in Wistar rats	*Mucuna pruriens L.*	Glibenclamide	Blood glucose level	Body weight loss associated with diabetes	Improve blood glucose level and body weight loss associated with diabetes	(p <0.001)	84 days	Low
32	2020	(38)	Tunisia	Experimental (*in vivo* animal study)	42 rats	42 Wistar rats	*Eruca sativa* leaves aqueous extracts	BPA (2,2-Di (4-hydroxyphenyl) propane)	Sperm parameters	Oxidative stress and testosterone levels	Improved sperm parameters, oxidative stress, and testosterone	(P<.05)	30 days	Low
33	2017	(39)	Nigeria	Experimental (*in vivo* animal study)	40 rats	40 STZ induced rats	Stem Bark of *Alstonia boonei*	Metaglomide	Oxidative stress parameters	Histology of the organs	Improved oxidative stress parameters and histological parameters	P<0.05	14 days	Low
34	2019	(40)	Pakistan	Experimental (*in vivo* animal study)	112 rats	male Sprague Dawley rats	*Ipomoea batatas L. Lam*	Sildenafil	levels of testosterone, FSH, LH, and Estradiol	Histomorphological examination of testes and oxidative stress parameters	Improved hormonal profile and histology of testes plus oxidative stress parameters	(P < 0.05)	21 days	Low
35	2025	(8)	Nigeria	Experimental (*in vivo* animal study)	24 rabbits	24 rabbit bucks	Ethanolic Bitter Kola (Garcinia kola)	Distilled water	Semen parameters	Testosterone levels	Improved sperm parameters and testosterone	(P < 0.05)	56 days	Low

Overall, Asian countries contributed approximately 45.71% of all included studies, highlighting their substantial contribution to research on diabetes and male reproductive health. Across all studies, commonly assessed biomarkers included glucose levels, testosterone levels, and oxidative stress biomarkers, including superoxide dismutase (SOD), glutathione peroxidase (GPx), catalase (CAT), and malondialdehyde (MDA). The majority of studies also evaluated sperm parameters and testicular histology, whereas only one study assessed offspring outcomes.

### Prevalence of interventions used for male fertility improvement

The most investigated plant extracts in this synthesis were used in two studies each (5.71% of all studies). These were as follows: *Mucuna pruriens* (Linn) leaf extract, purslane (*Portulaca oleracea*), chicory (*Cichorium intybus* L.), *Trifolium pratense* extract, and kolaviron (KV) extract from *Garcinia kola* (“bitter kola”). The other were evaluated in one study each (2.85%), including stem bark of *Alstonia boonei*, *Arctium lappa* (burdock) root, *Cochlospermum planchonii* rhizome, antioxidant icariside II, vincamine, *Securigera securidaca* seeds, and hesperetin flavonoids derived from orange peels.

The remaining plants were only used once in the studies, with the majority being aqueous extract of white tea (*Camellia sinensis*), *Gynura procumbens* leaf, *Hypoxis hemerocallidea* (HH) aqueous extract, soursop (*A. muricata*) tea, *Afzelia africana* (Smith), *Juglans regia* L. leaf extract, *Stevia rebaudiana Bertoni*, *Loranthus micranthus*, *Eruca sativa* leaf aqueous extract, *Ipomoea batatas* L. Lam, *Rosa canina*, *Mimosa pudica* Linn, *Momordica charantia*, green coffee arabica extract, porang (*Amorphophallus oncophyllus*), *Zataria multiflora* Boissis, and *Prunus amygdalas*, in addition to ethanolic extracts of *Bauhinia forficate* and *Launaea nudicaulis*, which were evaluated in 5.71% of studies.

### The use of diabetic animal models and population characteristics

More than 930 Wistar rats weighing between 120–250g were used in these studies, with the majority being male. However, three studies (8.57%) included a total of 120 female rats together with males for mating purposes. The most commonly used drug for inducing diabetes mellitus was streptozotocin (STZ) in doses ranging from 35mg/kg to 60mg/kg, and used in more than a third of the studies (34.29%). A total of 172 8-week-old Sprague-Dawley rats weighing 120–200 g were used in three studies (8.57%). In two of these studies, streptozotocin was also combined with nicotinamide to induce diabetes mellitus, and cisplatin was used to cause testicular toxicity.

The other drug that was used to induce diabetes in five studies (14.29%) was alloxan. It was administered to 143 male Wistar rats weighing between 150 and 200 g and to 48 alloxan-induced diabetic mice. The other 56 adult male mice were not induced, and neither were the 24 rabbit bucks.

### Comparator/control distribution and characteristics

Glibeclamide was the most commonly used comparator, appearing in seven studies (20%), followed by metformin in five studies (14.29%). Normal saline (2 ml/kg) (9%) and sildenafil citrate were each used in three studies (8.57%), while both distilled water and subcutaneous insulin were each used in two studies (5.71%). The remaining were only used in one study each as control (2.86%), including the HAART cocktail (zidovudine, lamivudine, and nevirapine), drinking water, metaglomide, BPA (2,2-Di (4-hydroxyphenyl) propane), cisplatin, clomiphene, tap water, streptozotocin, and etoposide.

### Prevalence of the key outcomes associated with hypoglycemic phytochemicals for male fertility

Improved blood sugar and hormonal profiles were the key outcomes in more than a third of the studies (34.29%). These were followed by sperm parameters, which were studied in 10 studies (28.57%). Oxidative stress was a key outcome in 8 studies (22.86%), followed by testicular histology parameters, which was evaluated in 7 studies (20%), of which 2 studies (5.71%) reported no improvement. Only 3 studies (8.57%) had testicular morphometric as the key outcome. The remaining outcomes were each reported in only one study (2.86%), including improvements in sexual behavior, glucose tolerance, erectile parameters, and libido score.

### SYRCLE risk of bias distribution and Grade framework

The majority of studies (29 of 35; 82.86%) had low risk of bias, showing no evidence of selection bias (sequence generation, baseline characteristics, or allocation concealment), performance bias (random housing or blinding), detection bias (random outcome assessment or blinding), attribution bias with complete outcome data, reporting bias, or other source of bias. Only 6 studies (17.14%) had challenges with attribution bias because of incomplete outcome data; thus, risk bias was unclear. Nevertheless, according to the GRADE framework, we did not downgrade the synthesis of the data used in these 35 primary publications.

## Discussion

This systematic review synthesized evidence from 35 preclinical *in vivo* studies evaluating the effect of hypoglycemic phytochemicals and plant extracts on the management of diabetes mellitus-induced male reproductive dysfunction. Across different animal models and geographic regions, the direction of effect was mostly consistent, indicating that hypoglycemic phytochemicals improved key reproductive endpoints, especially sperm parameters, testosterone levels, oxidative stress biomarkers, and testicular histological characteristics (8, 11, 27). Improved glycemic control was frequently noted together with reproductive benefits, indicating a close relationship between metabolic regulation and male reproductive function (20, 31).

The predominant reported outcomes were reduced fasting blood glucose and improved testosterone levels, each reported in approximately one-third of the included studies. Sperm quality parameters and oxidative stress markers were also improved (10, 36). Collectively, these results show that some hypoglycemic phytochemicals possess multisystem effects necessary to alleviate diabetes-associated male infertility challenges (13, 23, 33). The findings align with evidence associating chronic hyperglycemia, oxidative stress, and endocrine disruption with impaired spermatogenesis in DM (17, 18, 21).

**Figure 2:**
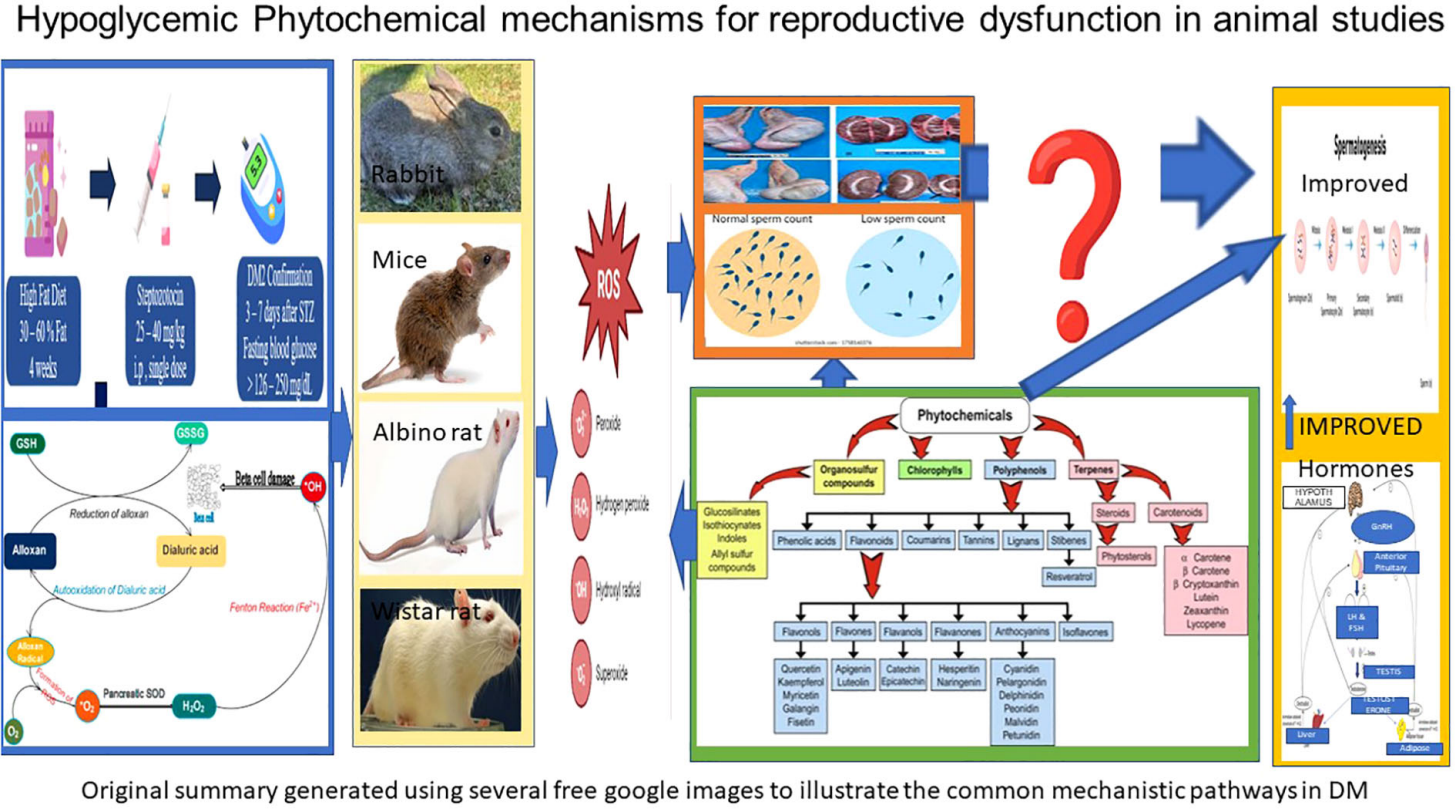
Mechanistic pathways for hypoglycemic phytochemicals in diabetes and infertility in animal experiments.

Previous *in vivo* studies have demonstrated that hyperglycemia leads to increased reactive oxygen species (ROS), which affect testicular mitochondrial function, compromise sperm membrane integrity, and suppress Leydig cell process of testosterone functions. The predominance of antioxidant and hormonal endpoints across studies suggests that glycemic control alone does not fully explain the observed fertility improvements. Rather, pleiotropic actions, including oxidative reduction modulation, as shown in **Figure 2**. anti-inflammatory effects, and hypothalamic pituitary gonadal axis regulation, likely contribute to the reproductive outcomes observed (16, 30, 39, 40).

Several phytochemical compounds have been shown in previous studies to have hypoglycemic activity. This is either through decreasing gluconeogenesis or increasing glycogenolysis and reducing Gastrointestinal tract (GIT) absorption (5). These hypoglycemic phytochemicals may also reduce oxidative stress through the reduction in end products of glycation and oxidative enzymes. The phytochemicals may affect α−amylase and α−glucosidase, as reported for the *Ficus* species, whose polyphenols are rich in flavonoids and phenolic acids. These agents lower the formation of advanced glycated compounds, which reduces the damage due to oxidative stress. In addition, several phytochemicals are known to directly increase antioxidant enzymes, such as catalase and superoxide dismutase. These upstream intracellular pathways reduce oxidative stress, apoptosis, and glycated cell injury due to diabetes mellitus (5, 37), which underscores their importance in DM-induced reproductive dysfunction.

The majority of studies used alloxan and STZ to induce diabetes mellitus by increasing oxidative stress in the pancreas, which destroys beta cells. This oxidative stress does not spare the reproductive system, especially the testes, which are sensitive to increased reactive oxygen species. Current hypoglycemic drugs have limited antioxidant properties to alleviate diabetes-induced reproductive dysfunction. Most plants used in the studies are known to be rich in flavonoids, polyphenols, alkaloids, and terpenoids, which possess antioxidant properties (25, 37, 41). The use of these hypoglycemic phytochemicals therefore offers dual therapeutic potential by improving plasma glucose levels while also alleviating reproductive dysfunction (32).

Several studies also reported reproductive improvements comparable to those of standard antidiabetic agents such as metformin and glibenclamide (15, 28, 29). Unlike some pharmacological agents that may have no known fertility benefits or even adverse reproductive effects, the phytochemicals reviewed generally showed reproductive safety and beneficial effects. This confirms the growing evidence that plant-derived hypoglycemic agents can offer both metabolic and reproductive advantages (14, 19, 34).

Nevertheless, despite the overall consistency in the direction of the reported effects, substantial heterogeneity was observed in the majority of studies. The sources of heterogeneity were due to the following: variation in animal species, use of streptozotocin versus alloxan in diabetic models, different phytochemical species and extracts, varying doses, and treatment duration. Outcome measures were also differently reported, especially for the sperm functional parameters and histological and morphological characteristics (6, 22, 26).

Quantitative synthesis was thus limited, and findings were synthesized narratively. However, appraisal using the SYRCLE Risk of Bias tool indicated that the majority of studies had a low risk of bias, particularly with respect to selection, performance, and detection biases. A few studies had unclear risk due to incomplete outcome data. According to the GRADE framework, the overall certainty of evidence was considered moderate, as the findings were consistent but derived only from preclinical models, consistent with the inclusion criteria (42).

From a translational perspective, the findings suggest that hypoglycemic phytochemicals have potential as complementary or alternative therapeutic strategies for diabetes-associated male infertility (11, 24). This is particularly relevant in low- and middle-income settings where traditional medicine use is prevalent and access to standard pharmacotherapy may be challenging (12), (35). However, this requires standardization of the phytochemicals and chronic toxicity studies to ensure efficacy.

Few studies evaluated fertility outcomes beyond sperm parameters, with only one study assessing offspring outcomes. In addition, there was limited standardization in phytochemical composition and dosing, reducing study reproducibility. Future research should focus on standardized extract characterization, longer intervention periods to assess offspring outcomes, including functional fertility outcomes, and detailed studies connecting metabolic and reproductive endpoints (42). Well-designed clinical trials will be required to characterize safety, efficacy, and optimal dosing in human clinical trials. This emphasizes the need for a coordinated, translational, and systems-level approach to addressing DM-related fertility complications. Standardizing preclinical study protocols for phytochemicals would improve study comparability and strengthen the quality of evidence for meta-analyses.

### Limitations

Study not being homogeneous: the included studies varied widely in preclinical model design, sample size, intervention duration, and outcome assessment, limiting the possibility of performing a meta-analysis.

Human diabetes mellitus, unlike animal models, contains psychological factors that may affect erectile function. This represents an important limitation in the translation of the preclinical studies to fully address the desired outcomes for improved reproductive dysfunction.

Publication bias: reporting mostly positive results may have overestimated the presumed beneficial effects of hypoglycemic phytochemicals and male fertility improvement interventions.

Long-term safety: apart from one study that evaluated offspring outcomes, limited evidence is available regarding the long-term safety and tolerability of these interventions, both of which are important for translating these interventions into daily use with confidence.

## Conclusion

The systematic review demonstrates that hypoglycemic phytochemicals and plant extracts have the potential to improve male reproductive dysfunction in preclinical models of diabetes mellitus. The synthesis highlights the beneficial effects on glycemic control, sperm quality, hormonal regulation, oxidative stress, and testicular histology via integrated metabolic and reproductive mechanisms. Although limited heterogeneity was present and the evidence was derived exclusively from animal studies, the main findings support the biological possibility and therapeutic potential of hypoglycemic phytochemicals for addressing diabetes mellitus-associated male infertility.

These results provide a good rationale for further *in vivo* studies and translational clinical research aimed at improving male reproductive health in patients with diabetes. Integrating preclinical findings with clinical research is essential, particularly given the widespread community use of plant extracts, to ensure safe and evidence-based translation into practice. At the policy level, DM programs should incorporate fertility screening and interventions to prevent reproductive complications. Strengthening research and clinical capacity is also critical to facilitate effective translation of preclinical evidence into sustainable clinical applications. In summary, community awareness programs should promote dietary diversification and safe supplementation practices to reduce fertility complications among patients with DM.

## Data Availability

The original contributions presented in the study are included in the article/**Supplementary Material**. Further inquiries can be directed to the corresponding author.
