# Supplementary figures and images for: The role of hypoglycemic phytochemicals in improving male reproductive dysfunction: a systematic review of preclinical evidence
Source: Front Endocrinol (Lausanne). 2026 Jul 13;17:1814324. doi: 10.3389/fendo.2026.1814324 (PMC13402205; doi:10.3389/fendo.2026.1814324)

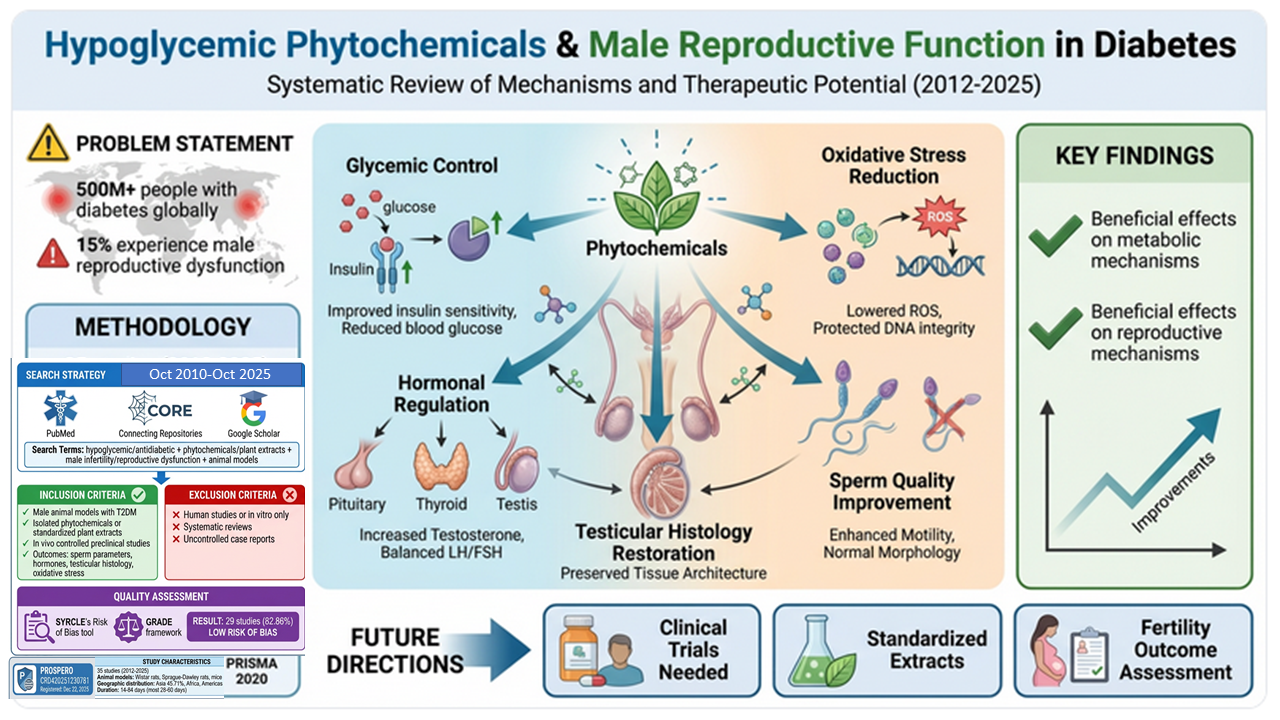

Supplement: Supplementary file 1 [file Image1.tif]
